# Supplementary figures and images for: A New Pipeline to Automatically Segment and Semi-Automatically Measure Bone Length on 3D Models Obtained by Computed Tomography
Source: Front Cell Dev Biol. 2021 Aug 26;9:736574. doi: 10.3389/fcell.2021.736574 (PMC8427701; doi:10.3389/fcell.2021.736574)

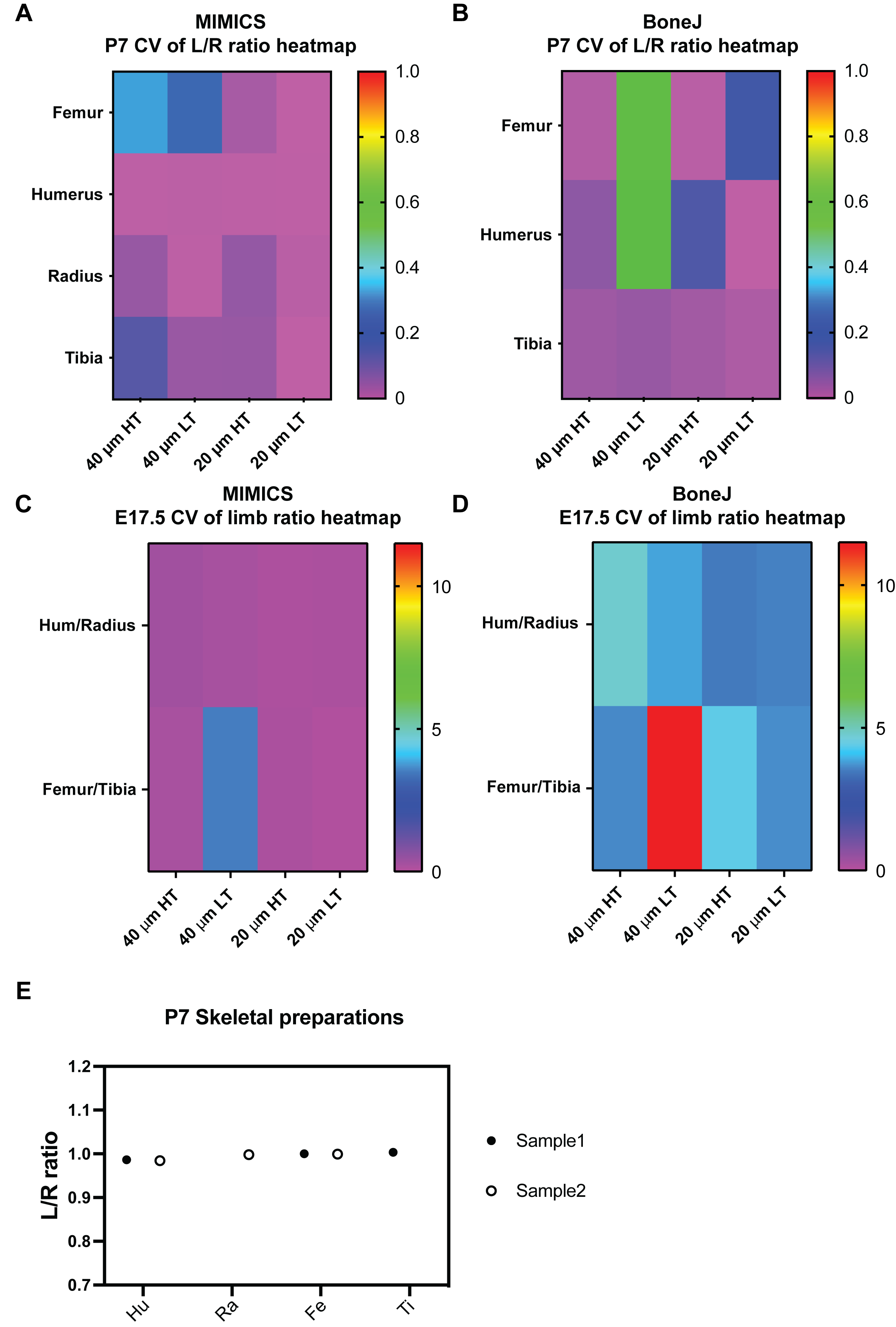

Supplement: Supplementary Figure 1 — (A) Representative 3D viewer result window after applying the Particle Analyser tool to a segmented and cleaned up whole-body scan (P7 mouse). (B–B″) Close-ups of femur (B), tibia [(B′), right] and humerus [(B′), left and (B″)] showing the features that can be obtained from the Particle Analyser tool, as indicated. Green arrowheads point to the ends of the maximum Feret’s diameter. The red arrowhead points to a region where the longest principal axis does not align with the skeletal element. (C) Two different samples were scanned four times at 40-μm resolution, and each of those scans were analyzed twice, with identical or nearly identical parameters. (D) Two different samples were scanned four times at 20-μm resolution, and most of those scans were analyzed once. (E) Heatmap with the average coefficient of variability for each bone and resolution. [file Image_1.TIF]

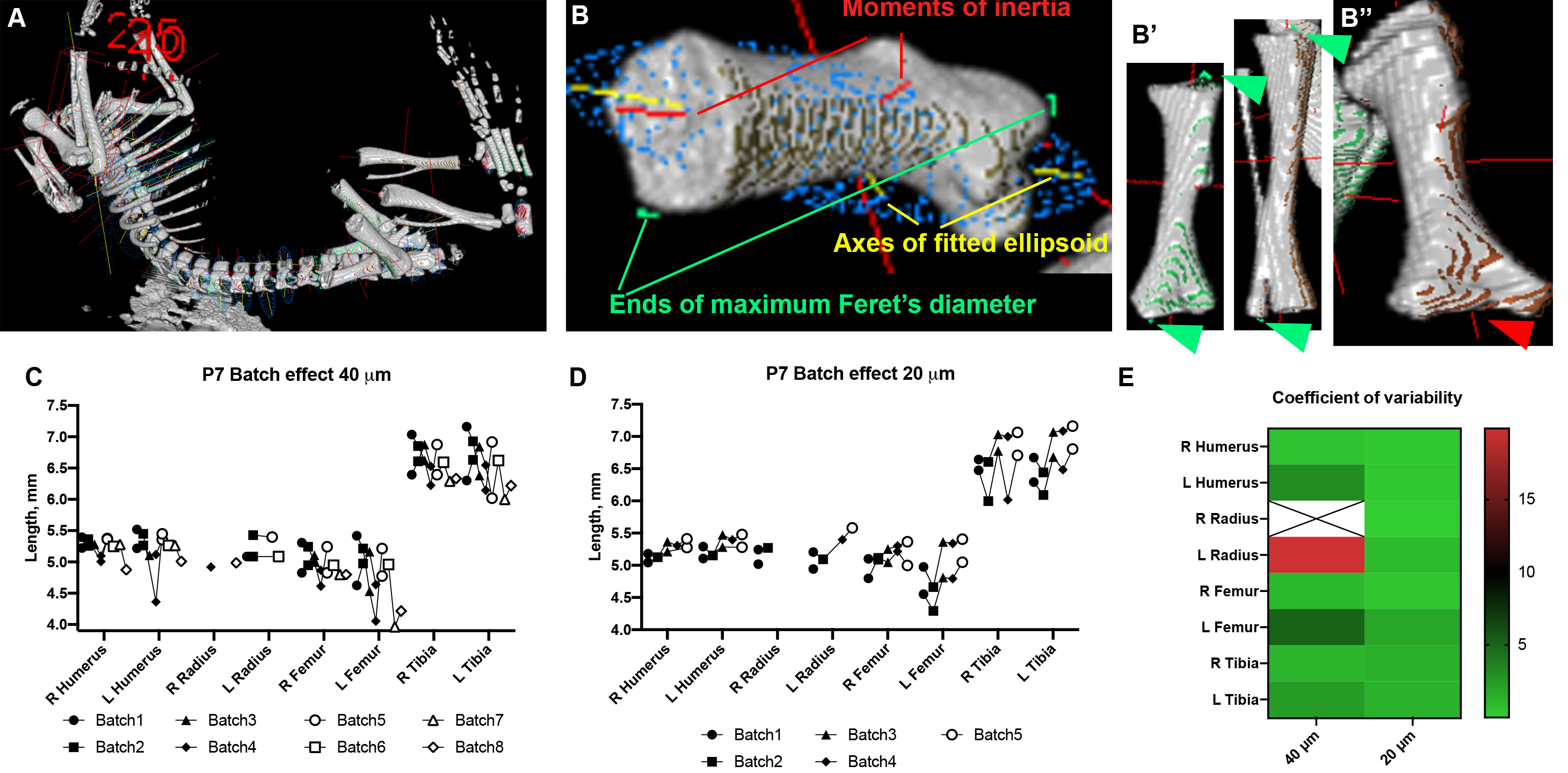

Supplement: Supplementary Figure 2 — Coefficients of variability for P7 and E17.5 analysis using Mimics and BoneJ. (A,B) Heatmaps for the CVs of the Left/Right length ratios obtained after analysis of P7 bones with Mimics (A) and BoneJ (B) pipelines. (C,D) Heatmaps for the CVs of the indicated ratios obtained after analysis of E17.5 bones with Mimics (C) and BoneJ (D) pipelines. (E) Left/Right ratios for the P7 bones used to benchmark BASILISC, measured after classic skeletal preparations. [file Image_2.TIFF]
